# Anæsthesia in Labor

**Published:** 1875-10

**Authors:** K. P. Moore

**Affiliations:** Knoxville, Crawford County, Georgia


ATLANTA
MEDICAL AND SURGICAL JOURNAL.

VOL. XIII.] OCTOBER—1875. [No. 7.

### Original Communications.

### ANÆSTHESIA IN LABOR.

BY K. P. MOORE, M.D., KNOXVILLE, CRAWFORD COUNTY, GA.

*Read before Middle Georgia Medical Society, July* 26, 1875.

Apropos of the discussion of anæsthesia in obstetrics in the last several meetings of the Academy of Medicine of Atlanta, as also a paper in ATLANTA MEDICAL AND SURGICAL JOURNAL for May, 1875, by B. H. Washington, M.D., on "Dry Cupping in Phlegmasia Dolens," I have thought it not inappropriate to prepare for our society a short paper on the subject of Anæsthesia in Labor.

Neither in the discussions above referred to, nor in Dr. Washington's paper, has any attempt been made at an explanation as to the *modus operandi* in which anæsthesia is beneficial in labor, other than relief from pain. It is true that mention was made by Dr. J. M. Johnson of rigid os or unyielding perineum, with violent pains, as cases or conditions indicating the use of anæsthesia, but *how* it was beneficial, or why the rigid os or unyielding perineum relaxed under its influence, without at the same time relaxing the fundus, remains yet to be explained. It is also true that Dr. Washington, in his paper on phlegmasia dolens, has told us that dry cupping on the spinal column would relax the rigid os and external parts, while it would bring on contractions of the fundus; but without some further explanation as to how this was *probably* accomplished, his views might be considered, as a medical friend expressed it to me while discussing the article, "too fine spun." It is an attempt at an elucidation of this subject that prompts the inditement of this paper.

It may seem a little presumptuous in one of my age to attempt an elucidation of a subject which has just been handled by men of long experience and extended observation and research; but I do not wish to be understood as assuming to myself any superior knowledge upon this subject, nor of presenting anything new to the gentlemen of the Academy or Dr. Washington, but of calling attention to what they seem to have overlooked, and which is necessary to strengthen Dr. Washington's paper, as also Drs. J. M. Johnson, Taliaferro and others who have timidly confessed that they could be persuaded under very trying circumstances to use chloroform in labor, (the verdict of a Boston jury to the contrary notwithstanding.)

I have always been a firm advocate for anæsthesia in labor, and have bestowed no little thought upon the subject from my first case until now; and though I have made almost an habitual use of it, I have yet to see the first untoward symptom resulting from it; and were I a woman, and my accoucheur refused to give me chloroform, other things being equal, I should at least suggest the propriety of getting another "granny." Of course every condition which would otherwise contraindicate its use should be looked well into, and exceeding caution observed. I always watch its effect upon the circulation and respiration especially.

I have never used any other agent in obstetrics than chloroform, though it requires some little courage to acknowledge it in these times of extreme fashion in medicine. Just here I will venture the belief that the scare and fright from chloroform, (which reminds one of a drove of black birds being shot at in a wheat patch) is more of fashion than anything else. We are taught that chloroform is a powerful brain sedative; that the brains of those who die from chloroform have been found to be almost free from blood. And we know, as J. Marion Sims expresses it, "the efforts of parturition cause cerebral congestion, which counteracts the tendency to syncope from chloroform."

To come more directly to the subject, we propose to give our idea as to how chloroform hastens labor, or how dry cupping may possibly have the effect ascribed to it by Dr. Washington. We confess that reason doesn't seem to impress us with as much confidence in the remedy proposed by Dr. Washington as his experience does him; yet we might be otherwise taught by experiment, and we think it fully worth while trying it, and hope to hear from others. The great ganglionic or sympathetic system of nerves, it will be remembered, preside over those organs over which the consciousness and the will have no immediate control, and that the organs thus supplied are much less susceptible to external impressions than those supplied by the cerebro-spinal system. It will also be remembered that they respond much more slowly to stimuli, and are much more slowly affected by narcotics or anæsthesia than those under the control of the cerebro-spinal system. This is demonstrated to us in our every-day observation. Who does not know that our involuntary and more vital organs are the last to succumb to the influence of an anæsthetic or narcotic? If this were not the case we could not use anæsthesia at all; for if the sympathetic system, and the organs supplied by it, were equally and as easily brought under the influence of anæsthetics as those under the cerebro-spinal system, we might expect the heart, which is almost wholly supplied by the sympathetic, to become anæsthetized as soon as any other muscle; but to the contrary we find the heart to be the last to yield to its influence. So with all the organs, in proportion to the supply of influence from the sympathetic system. Even in those organs supplied from both systems, the response to stimulus is much more sluggish than where there is no sympathetic distributions. This can be better illustrated in the case of the eye, by the stimulating influence of light over the alternate expansion and contractions of the pupil.

"The ophthalmic ganglion sends off a number of ciliary nerves, which are distributed to the iris; receiving also a sensative root from the ophthalmic branch of the fifth pair, and a motor root from the oculo-motorius." The reflex movements of the iris exhibit consequently a somewhat sluggish character, caused by the intervention of sympathetic influence.

The changes in the size of the pupil do not take place instantaneously with the variation in the amount of light, but always require an appreciable interval of time. If we pass suddenly from a brilliantly lighted apartment into a dark room, we are unable to distinguish surrounding objects until a certain time has elapsed, and the expansion of the pupil has taken place; and vision continues to grow more and more distinct for a considerable period afterward, as the expansion of the pupil becomes more complete.

Furthermore, if we pass from a dark room into a bright sunshine, we are immediately conscious of a painful sensation in the eye, which lasts for a considerable length of time, and which results from the inability of the pupil to contract with sufficient rapidity to shut out the excessive amount of light. This shows that even when an organ is supplied from both systems, that the sympathetic counteracts the rapid action which the cerebro-spinal would otherwise exert.

Now we find that the uterus throughout its whole extent, and especially the fundus, is largely under the control of the hypogastric ganglia of the sympathetic system. Indeed the gravid uterus is almost entirely under its influence, as we learn from Dr. Robert Lee's beautiful anatomy of the nerves of the gravid uterus. The cervix and vagina are supplied from both systems, but in the vaginal canal the cerebro-spinal nerves predominate, and as we approach the external opening we find the sphincter vaginæ and perineal muscles entirely under the control of the cerebro-spinal system.

We have already shown that the spinal system is much more susceptible to anæsthesia than the sympathetic, and indeed may for some time become anæsthetized before the sympathetic is at all, or but slightly affected; hence as the cervix and vaginal canal are largely supplied from the cerebro-spinal, and the external vaginal and perineal muscles wholly supplied from it, while the fundus of the gravid uterus is almost or entirely under the influence of the sympathetic, we can easily see how anæsthesia would not interfere with the contractions of the uterus, unless long continued, while it would cause relaxation of the cervix and external genitals.

It is not my purpose to speak of the manner in which I use it. I have accomplished the object sought in the paper. But some one may want to know my plan, and I will briefly state it. I rarely give it in the first stage of labor, but begin with the second stage, and give it moderately until labor is over. Rarely ever carry it to complete anæsthesia, but keep my patient sufficiently under its influence to sleep during the intervals between pains. Have often had them to express themselves as passing through the most trying ordeal of a woman's life "on flowery beds of ease." Were we called to a *man* who was compelled to suffer for the same length of time half the pain usually endured by women, we would not hesitate to do all in our power to relieve him: why then withhold it from—

"Woman, dear woman, whose form and whose soul
Is the life and the light of each spell we pursue;
Whether sunned at the tropics or chilled at the pole,
If woman be there, there is happiness too."

